# When It’s Not Worn on the Face: Trait Anxiety and Attention to Neutral Faces Semantically Linked to Threat

**DOI:** 10.3390/vision8010015

**Published:** 2024-03-19

**Authors:** Kim M. Curby, Jessica A. Collins

**Affiliations:** 1 School of Psychological Sciences, Macquarie University, Sydney, NSW 2109, Australia; 2 Biogen, Cambridge, MA 02142, USA

**Keywords:** semantic knowledge, faces, emotion, attention, trait anxiety

## Abstract

While our direct observations of the features or behaviours of the stimuli around us tell us much about them (e.g., should they be feared?), the origin of much of our knowledge is often untethered from directly observable properties (e.g., through what we have learned or have been told about them, or “semantic knowledge”). Here, we ask whether otherwise neutral visual stimuli that participants learn to associate with emotional qualities in the lab cause the stimuli to be attended in a similar way as stimuli whose emotional qualities can be discerned through their visual properties. In Experiment 1, participants learned to associate negative or neutral characteristics with neutral faces, which then served as valid or invalid spatial cues to targets in an attentional disengagement paradigm. The performance of participants higher in trait anxiety was consistent with attentional avoidance of faces with learned negative associations, while participants lower in trait anxiety showed a general response slowing in trials with these stimuli, compared to those with neutral associations. In contrast, in Experiment 2, using (visually) expressive (angry) faces, the performance of participants higher in trait anxiety was consistent with difficulty disengaging from visually threatening faces, while the performance of those with lower trait anxiety appeared unaffected by the valence of the stimuli. These findings suggest that (1) emotionality acquired indirectly via learned semantic knowledge impacts how attention is allocated to face stimuli, and this impact is influenced by trait anxiety, and (2) there are differences in the effects of stimulus emotionality depending on whether it is acquired indirectly or directly via the perceptual features of the stimulus. These differences are discussed in the context of the variability of attention bias effects reported in the literature and the time course of impacts of emotionality on stimulus processing.

## 1. Introduction

Our visual world is imbued with meaning, with many of the objects we encounter having a rich set of associated semantic knowledge. Semantic knowledge can convey much of the same information about an object as that which can be acquired through direct interaction. For example, while an individual might develop a fear of dogs after interacting with an aggressive dog, another individual may become fearful of Doberman Pinschers after reading about their potentially aggressive nature. There is an abundance of evidence demonstrating the prioritisation of visually threatening stimuli, such as angry faces, particularly amongst anxious groups (see [1] for a review). However, an open question is whether stimuli with emotional associations, such as fear acquired through abstract knowledge, are similarly prioritised for processing as are visually emotional stimuli.

Previous research has demonstrated that learned semantic knowledge can impact how an object is perceived, even when it is task-irrelevant. For example, the matching of two-dimensional or three-dimensional objects is facilitated when those objects are associated with distinctive semantic concepts as opposed to semantic concepts that are similar to each other [2]. Additionally, training participants to associate arbitrary semantic information with novel objects served to reduce the cost to recognition performance typically incurred when an object is presented from a novel viewpoint [3,4]. Electrophysiological research has also demonstrated a larger P1 amplitude for novel objects that were associated with minimal knowledge relative to objects that were associated with in-depth semantic knowledge [5]. Notably, the P1 is considered a marker of early perceptual processing [6,7]. The reduction in its amplitude was interpreted by the authors as reflecting reduced demands on visual processing and thus more efficient visual processing of stimuli with rich semantic associations. Together, these studies suggest an ability of stored semantic knowledge to shape relatively early stages of visual processing.

Notably, emotionality derived from the visual aspects of a stimulus has also been shown to measurably affect perceptual processing. For example, in a visual search task where participants search for a target among an array of distracters, participants were faster at detecting an angry target face than a neutral or happy one [8,9]. Additionally, in the classic attentional blink paradigm, where responding to a target in a rapid stream of items causes participants to “blink” or miss a second target if it appears shortly after the first one, people were less likely to miss an angry face, a neutral face conditioned with a loud burst of noise, or an emotional word [10,11,12]. These findings suggest that emotionality can increase the salience of a stimulus.

Emotion can also impact the processing of objects indirectly, with the visual processing of non-emotional objects influenced by the emotional context in which they are presented. For example, in a visual search task, participants more quickly located a target image (a house, bird, or car) when an angry face (compared to a happy or neutral face) preceded the search array [13]. Also, the presentation of an angry, relative to a neutral, face cue has been found to reduce the amount of visual contrast necessary to perform a simple discrimination task with a non-face visual target [14]. Thus, there is evidence that emotion can influence the processes underlying the perception of stimuli both directly and indirectly.

The impact of emotional salience on visual perception can be mediated by individual differences in trait anxiety. For example, several forms of social attention are influenced by an individual’s level of anxiety and the emotional content of stimuli, including attention holding [15], attention cueing [16], and attentional orienting [17]. Specifically, using the dot probe task, in which neutral and negative stimuli appear simultaneously, with one then replaced by a target, Bradley and colleagues [18] found that people were faster to respond to the target when it replaced a threat-related, rather than neutral, cue. Further, this effect was greater among people with high levels of trait anxiety.

Notably, the dot probe paradigm has yielded inconsistent results with respect to the patterns of performance of anxious individuals (see [19] for a review). While a systematic review supported the existence of a preference to allocate attention towards threat-related, compared to neutral, faces amongst socially anxious individuals, the pattern of performance in this task appears to depend on a number of task parameters, including the type of reference stimulus, the stimulus duration, and the level of anxiety of the participants [19]. The heterogeneity in findings using this paradigm suggest that further research is required to better understand the factors that determine how trait anxiety impacts attention to threatening stimuli.

One issue with the standard dot probe paradigm is that because the threat-related and neutral cues are presented simultaneously in this paradigm, it is not clear whether the facilitated target perception in the high-anxiety participants is due to enhanced orienting towards the valid threatening cue or to an increased attentional dwell time on the threatening cue. To address this issue, Fox and colleagues [20] developed an attentional disengagement task with the goal of teasing apart potentially enhanced orienting towards, versus increased dwell time on, threatening stimuli amongst participants high in trait anxiety. In this paradigm, one spatial cue is presented on either side of a central fixation and is followed by a target in either the same location as the cue (valid cue condition) or the opposite location from the cue (invalid cue condition). Using angry, neutral, or happy schematic faces as valid or invalid spatial cues, Fox and colleagues [20] found that participants with high trait anxiety, but not low trait anxiety, demonstrated greater difficulty disengaging their attention from an angry face than from a neutral or happy face. This effect has been replicated using images of real emotional faces [21], neutral and emotional word stimuli [22], and threatening and neutral scenes [23,24].

Although substantial research has investigated the visual perception of emotional stimuli (such as angry faces), little work has addressed if or how conceptual emotional associations influence visual processing. This is an important gap in the literature as the semantic knowledge associated with stimuli, such as faces, often has an emotional tone. The goal of the current study was to investigate whether emotional meaning derived through semantic learning impacts perception in a manner similar to that previously reported for visually emotional stimuli. In other words, does a person need to look angry for their face to be preferentially processed, or might having knowledge of that person’s angry disposition drive a similar impact on perceptual processing? To investigate this question, participants were trained to associate negative or neutral emotional associations with a set of visually neutral face images. Visual processing of these faces was then assessed using an attentional disengagement paradigm [20]. We hypothesized that neutral faces with learned negative associations would hold attention longer than neutral faces amongst participants with higher trait anxiety.

## 2. Experiment 1

### 2.1. Method

Participants: A total of 117 introductory psychology undergraduate students (70% female) completed this study for course credit. The final sample size was 109 after the inclusion criteria were applied (see results for details). A post hoc power analysis revealed that this sample provided a power level of 0.91, assuming a medium effect size (η_p_ = 0.06). The participants had a mean age of 20.00 years (*SD* = 2.44) (Demographic information is only available for 84 of the participants. However, given the typical characteristics of first-year psychology undergraduate participant pools in terms of age and gender, we expect that this mean should approximate the mean of the full sample.). All participants reported having normal or corrected-to-normal vision. Informed consent was obtained from all subjects involved in this study. This study was conducted in accordance with the Declaration of Helsinki and approved by the Temple University Institutional Review Board. This study was not preregistered.

Materials: Eight Caucasian male faces with neutral expressions were selected for this experiment (from [25]). Each face was cropped in Photoshop and placed within an oval frame so that no hair was visible. For the attention disengagement paradigm, the faces were presented within dark grey rectangles that were 5.3 cm high and 3.0 cm wide. The rectangles were placed so that the closest edge of each rectangle was 2.0 cm from each side of a central fixation point. The target images consisted of a small white square with a diameter of 0.3 cm or a small white circle with a diameter of 0.3 cm. Each of these target images was presented within the dark grey boxes.

Procedure: The experiment began with a study phase in which subjects learned to associate three personality characteristics with each of the eight faces. The sets of characteristics could either be non-threatening (neutral) in valence (e.g., “busy, curious and quick”), or threatening (negative) (e.g., “angry, violent and unpredictable”), with each characteristic being associated with only one of the facial stimuli (see Appendix A for the full list of characteristics). The neutral and negative lists were comparable in terms of word frequency (*p* = 0.25) and number of syllables (*p* = 0.36). The assignment of each face to a valence category was counterbalanced across participants. By counterbalancing which faces had learned negative associations and which faces had learned neutral associations, any differences in the responses to these faces could be attributed to the learned emotional valence of the stimuli and not to differences in their perceptual salience. During the first phase of training, the participants were shown each of the eight faces four times simultaneously with two characteristics from their descriptive set for 5 s. The participants then answered 32 questions to probe their memory for the two characteristics that they had just learned for each face (e.g., “Is this person angry and violent?”). Following this, four of the faces (two with negative associations and two with neutral associations) were each shown six times simultaneously with all three characteristics from their descriptive set for 5 s. The participants then answered 48 questions about the previously displayed four faces and single characteristics (e.g., “Is this person busy?”). Afterwards, the remaining four faces were each displayed six times simultaneously with all three characteristics from their descriptive set for 5 s. The participants then answered 100 questions about a single characteristic (e.g., “Is this person violent?”) in which all eight faces appeared to probe their memory for the associations. To facilitate learning, at six points during the training, the participants wrote down all the characteristics they could remember for each of the facial stimuli. To assess learning, the participants performed matching trials in which one of the sets of three characteristics were displayed at the top of the screen while 6 of the faces were displayed below with associated numeric responses (1–6); participants indicated via button press which face was associated with the given set of characteristics. Each block consisted of 48 trials. Accuracy was assessed at the end of each block, with participants passing the training criteria if they accurately completed at least 75% of trials in a block for both the neutral and negative semantic training conditions.

**Figure 1:**
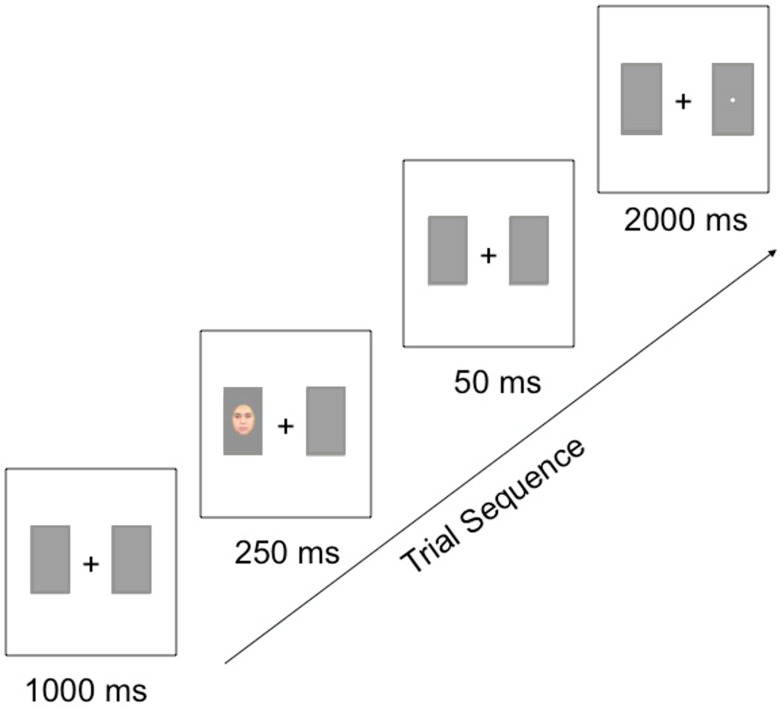
Trial sequence for the attentional cueing task used in Experiments 1 and 2.

Following the study phase, subjects completed the attentional disengagement task in which the learned faces served as spatially valid or invalid cues to a target image. A visual schematic of this paradigm is displayed in Figure 1. In each trial, the participant was first presented with a central fixation (+) surrounded on each side by two grey boxes for 1000 ms. One of the faces from the training paradigm was then presented in one of the grey boxes for 250 ms, and then removed for 50 ms. The participant’s task was to categorize a target image, either a small square or circle that then appeared on either the right- or left-hand side of the central fixation for 2000 ms or until the participant responded. All participants responded by pressing “1” when the target was a square, and “2” when the target was a circle. Participants were instructed to fixate their gaze on the central fixation and to categorize the target image as quickly as possible without sacrificing any accuracy. In 75% of the trials, the face served as a valid spatial cue, with the target image occurring in the same location as the face. In the remaining 25% of the trials, the target image occurred in the opposite location to the face. Participants completed 30 practice trials, followed by four blocks of 64 trials each, resulting in 256 experimental trials in total. Each facial stimulus appeared as an invalid spatial cue 8 times (four times on either side of the central fixation) and as a valid cue 48 times (24 times on either side of the central fixation). Each target type (square or circle) appeared equally often in each condition of the experiment. The order of trials was randomized within each block for each participant.

Following the attentional disengagement task, participants filled out the State Trait Anxiety Inventory (STAI; [26]). All items are rated on a 4-point scale (e.g., from “Almost Never” to “Almost Always”). Higher scores indicate greater anxiety with the range of possible scores from 20 to 80 on both the STAI-T (20 questions) and STAI-S (20 questions) subscales. Spielberger et al. [26] suggest that 20–39, 40–59, and 60–80 indicate low, moderate, and high anxiety, respectively. Although the specific cut-offs for lower and higher anxiety groups used in studies with non-clinical populations varies, the means for the lower anxiety groups tend to be in the thirties or lower, while those for the higher anxiety groups tend to be in the forties or above (e.g., [27]).

### 2.2. Results

Trials with incorrect responses and RT latencies below 200 ms or above 1000 ms were removed from the data. In addition, participants with fewer than 85% of their data remaining after these filters were applied (*N* = 8) were removed. An average of 5.9% (*SD* = 3.7%) of the data was excluded from the remaining participants (1.9% as a result of the response time filter and 4.0% due to inaccurate responses).

Following procedures used in previous studies, participants were then assigned to lower- and higher-trait-anxiety groups using a median split of the STAI Trait Anxiety scores (e.g., [28]); participants scoring 41 or below were assigned to the lower-trait-anxiety group (*N* = 58; *M* = 36.5, *SD* = 3.4), and those scoring > 41 were assigned to the higher-trait-anxiety group (*N* = 51; *M* = 48.5, *SD* = 6.0). Note that the number of participants assigned to each group differed due to the participants scoring 41 being assigned to the lower anxiety group. The number of additional test blocks required for high (*M* = 1.041, *SD* = 0.41 blocks) versus low (*M* = 1.037, *SD* = 0.48 blocks) anxious participants to reach the criterion during the semantic association training phase was well matched.

A two (trait anxiety) × two (cue validity) × two (cue valence) ANOVA performed on the RT data from correct trials revealed a significant main effect of cue validity (*F*(1,107) = 318.96, *p* < 0.001, η_p_^2^ = 0.75), with participants responding more quickly in valid than invalid trials. There was no main effect of cue valence (*F*(1,107)= 2.39, *p* = 0.13, η_p_^2^ = 0.022) or trait anxiety grouping (*F*(1,107) = 3.15, *p* = 0.079, η_p_^2^ = 0.029). There were also no significant two-way interactions between any of the variables (all *p*s > 0.11). However, there was a significant trait anxiety × cue validity × cue valence interaction (*F*(1,107) = 7.22 *p* = 0.0084, η_p_^2^ = 0.063).

**Figure 2:**
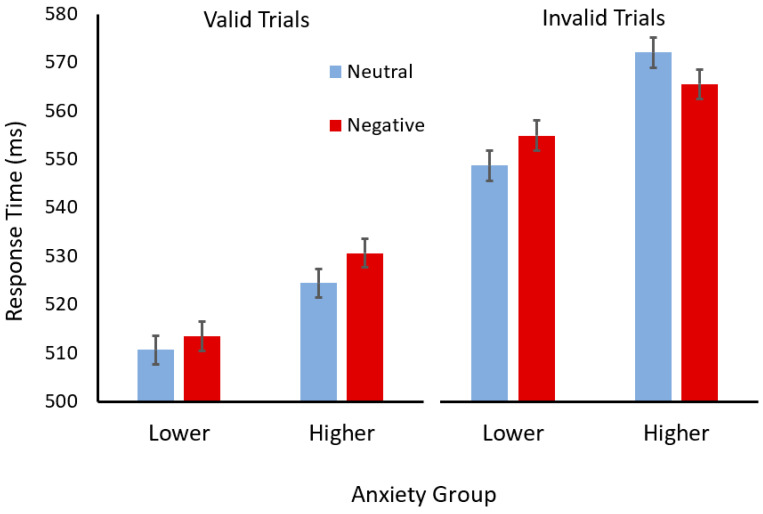
Mean response times (ms) for valid (left) and invalid (right) trials with face cues with neutral (light blue) or negative (dark red) semantic associations for participants in the lower- and higher-trait-anxiety groups. Error bars show standard error of the mean.

To break down this interaction, the results for participants in each anxiety group were analysed separately (see Figure 2). For the participants with higher trait anxiety, a two (cue validity) × two (cue valence) ANOVA revealed a significant main effect of validity (*F*(1,50) = 154.09, *p* < 0.001, η_p_^2^ = 0.76) but not valence (*F* < 1). However, there was an interaction between validity and valence (*F*(1,50) = 8.18, *p* = 0.0006, η_p_^2^ = 0.14). Scheffé tests revealed that participants with higher trait anxiety responded faster in the invalid trials when the face cue had a negative, compared to neutral, learned valence (*p* = 0.046). In contrast, they appeared to respond *slower* in valid trials when the face cue had a negative learned valence, although this difference was marginal (*p* < 0.051).

For the participants with lower trait anxiety, a two (cue validity) × two (cue valence) ANOVA revealed a significant main effect of validity (*F*(1,57) = 165.62, *p* < 0.001, η_p_^2^ = 0.74), with participants responding faster after valid, than invalid, cues. In addition, there was a main effect of valence (*F*(1,57) = 4.65, *p* = 0.035, η_p_^2^ = 0.075), with participants responding more slowly after face cues with negative, compared to neutral, associations. The interaction between cue validity and cue valence was not significant (*F* < 1).

### 2.3. Discussion

Our findings suggest that emotional meaning acquired through semantic learning can impact how attention is allocated to face stimuli. However, when considered in the context of previous findings, the current data suggest that the impact of trait anxiety on such effects may differ depending on whether the emotional properties of the stimulus are derived directly (i.e., visually) or via learned semantic associations. Specifically, while previous studies using visually emotional cues (e.g., schematic faces with threatening expressions) reported high trait anxiety to be associated with *slower* disengagement from emotional stimuli, we found that participants higher in trait anxiety were *faster* to disengage from invalid face cues with learned negative, compared to neutral, semantic associations. Together with the trend suggesting that they were also slower to orient to valid faces cue with negative, compared to neutral, associations, this finding suggests that learned negative semantic associations may trigger an avoidance response amongst participants higher in trait anxiety.

## 3. Experiment 2

The apparent attentional avoidance response to faces with learned negative associations observed amongst participants higher in trait anxiety in Experiment 1 appears inconsistent with several previous studies using visually emotional faces. These studies found that participants higher in trait anxiety demonstrated difficulty disengaging attention from such stimuli [20]. However, several other studies have found that both patterns of attentional avoidance and prioritisation of (and difficulty disengaging from) emotional stimuli can be observed depending on the paradigm used and the parameters chosen. For example, attentional prioritisation and difficulty disengaging from emotional stimuli is often seen for shorter presentation durations, while longer presentation durations have been shown to result in patterns of attentional avoidance (e.g., [29,30,31]). Notably, there are methodological differences between the current study and these previous studies using visually emotional stimuli, other than those central to our training manipulation (e.g., some differ in the stimuli used and others in the presentation time of the face stimuli). These differences limit the insights that can be made based on direct comparisons between the studies. Thus, it is an open question as to whether visually emotional faces (i.e., those where the emotionality could be observed directly rather than learned through association) would also produce an attentional avoidance response amongst participants higher in trait anxiety if the same paradigm and parameters were used as in Experiment 1. The purpose of Experiment 2 was to repeat Experiment 1 using visually emotional (angry) faces to better elucidate whether perceptual and semantic emotional meaning have a similar or distinct impact on the orienting of attention.

### 3.1. Method

Participants: A total of 115 undergraduate introductory psychology students (70% female) completed this study for course credit. The participants had a mean age of 20.64 years (*SD* = 3.6) and had normal or corrected-to-normal vision. Informed consent was obtained from all subjects involved in this study. The final sample size was 96 after the inclusion criteria were applied (see methods for details). A post hoc power analysis revealed that this sample provided a power level of 0.87, assuming a medium effect size (η_p_ = 0.06). This study was conducted in accordance with the Declaration of Helsinki and approved by the Temple University Institutional Review Board. This study was not preregistered.

Materials: Eight Caucasian male faces, four with angry expressions and four with neutral expressions, were selected for this experiment from the NimStim Set of Facial Expressions ([32]; available at http://www.macbrain.org/faces). Each face was cropped in Photoshop so that all hair was removed. For the attention disengagement paradigm, the faces were presented within dark grey boxes that were 5.3 cm high and 3.0 cm wide and were displayed 2.0 cm to the left and right of the central fixation point. The target images consisted of a small white square with a diameter of 0.3 cm or a small white circle with a diameter of 0.3 cm. Each of these target images was also presented within the dark grey boxes.

Procedure: Participants completed the same attentional disengagement procedure as outlined in Experiment 1. Following the attentional disengagement procedure, participants filled out both the state and trait versions of the State Trait Anxiety Inventory (STAI; [26]).

Data processing: One participant was excluded as they exceeded the age inclusion criteria listed on the ethics approval (>40 years). Following the same procedure as for Experiment 1, incorrect responses and RT latencies < 200 ms or >1000 ms were removed from the data. Performance in general was lower in Experiment 2, with the data from 18 participants excluded after the same requirement as Experiment 1 was applied; that is, participants must have >85% of their data remaining. An average total of 6.3% (*SD* = 3.5%) of data was excluded from the remaining participants (1.9% as a result of the response time filter and 4.4% due to inaccurate responses). The remaining participants were assigned to low- and high-trait-anxiety groups using a median split; participants scoring below 47 were assigned to the low-trait-anxiety group (*N* = 44; *M* = 34.16, *SD* = 6.36), and those scoring 47 or higher were assigned to the high-trait-anxiety group (*N* = 52; *M* = 51.29, *SD* = 4.33). Given that the median trait anxiety score was higher in Experiment 2 than in Experiment 1, participants with the median score were assigned to the high-trait-anxiety group so the mean scores for the high- and low-trait-anxiety groups in Experiment 1 and 2 were more closely matched.

### 3.2. Results

The RT data for correct trials were subjected to a two (trait anxiety group) × two (cue validity) × two (cue valence) ANOVA. There was a significant main effect of cue validity (*F*(1,94) = 197.76, *p* < 0.001, η_p_^2^ = 0.68), with participants responding more quickly in valid than invalid trials, but no main effect of cue valence was observed (*F*(1,94)= 2.16, *p* = 0.15, η_p_^2^ = 0.022). However, there was an interaction between cue validity and cue valence (*F*(1,94)= 4.34, *p* = 0.04, η_p_^2^ = 0.04). Scheffé tests revealed that participants responded slower following facial cues with angry expressions relative to neutral expressions in invalid (*p* = 0.02) but not valid (*p* = 0.6) trials. There was also a marginal three-way interaction between trait anxiety level × cue validity × cue valence (*F*(1,94) = 3.86, *p* = 0.05, η_p_^2^ = 0.04).

**Figure 3:**
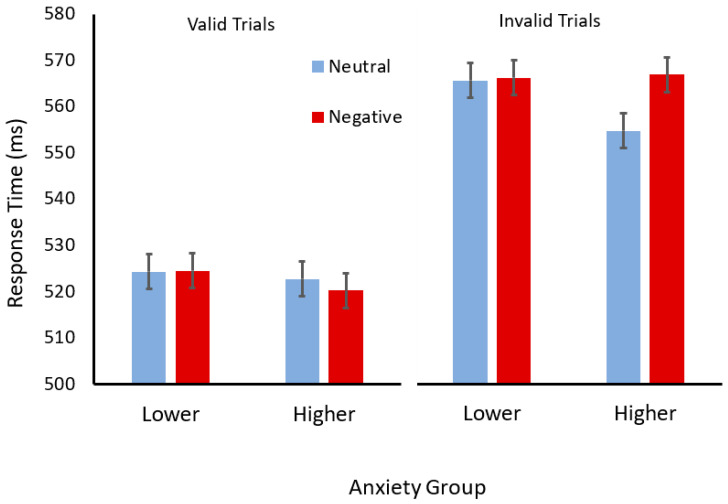
Mean response times (ms) for valid (left) and invalid (right) trials with face cues with neutral (light blue) or negative (dark red) expressions for participants in the lower- and higher-trait-anxiety groups. Error bars show standard error of the mean.

For consistency, the same analyses performed for Experiment 1, separating the anxiety groups, were performed (see Figure 3). For the participants with higher trait anxiety, a two (cue validity) × two (cue valence) ANOVA revealed a significant main effect of validity (*F*(1,51) = 114.16, *p* < 0.001, η_p_^2^ = 0.69) but not valence (*F*(1,51) = 3.76, *p* = 0.06, η_p_^2^ = 0.07). However, there was an interaction between validity and valence (*F*(1,51) = 9.44, *p* = 0.003, η_p_^2^ = 0.15). Scheffé tests revealed that participants with higher trait anxiety responded slower after negatively valenced (angry) compared to neutral cues, but only when these cues were invalid (*p* < 0.001) and not when they were valid (*p* = 0.45). For the participants with lower trait anxiety, a two (cue validity) × two (cue valence) ANOVA revealed a significant main effect of validity (*F*(1,43) = 85.57, *p* < 0.0001, η_p_^2^ = 0.66) but no main effect of valence (*F* < 1), and no interaction between cue validity and cue valence was observed (*F* < 1).

Matching trait anxiety group means across Experiments 1 and 2: To aid the comparison of the findings of Experiment 2 with those from Experiment 1, the data from Experiment 1 were reanalysed after the mean trait anxiety scores of the lower and higher anxiety groups were matched with those in the current experiment. This required removing participants with the lowest trait anxiety scores from the high anxiety group and those with the highest trait anxiety scores from the lower anxiety group from Experiment 1. We chose to match to the means from Experiment 2, as Experiment 1 had a large initial sample size, so removing participants to match the means would also lead to a closer matching of sample sizes. In addition, there was a bigger difference between the means in the lower- and higher-trait-anxiety groups in Experiment 2; thus, reducing this difference to match the means with Experiment 1 would reduce the ability to see a trait anxiety-related difference between the groups. Participants were removed in order (most extreme scores first) until the mean trait anxiety scores were no longer statistically different between the groups in Experiments 1 and 2 (low-trait-anxiety group, *M* = 36.04, *SD* = 3.30; *p* = 0.06; high-trait-anxiety group, *M* = 49.38, *SD* = 5.95; *p* = 0.08). The data from ninety-eight participants remained. The analysis of the remaining participants’ data was then performed. This analysis revealed the same general pattern of findings as the original analysis. That is, there was an interaction between the trait anxiety group, cue valence, and cue validity (*F*(1,96) = 6.44, *p* = 0.013, η_p_^2^ = 0.063). There was also a marginally significant interaction between cue valence and cue validity (*F*(1,96) = 3.95, *p* = 0.05, η_p_^2^ = 0.040). The main effect of anxiety group also reached significance, with the higher anxiety group generally responding more slowly (*F*(1,96) = 6.79, *p* = 0.011, η_p_^2^ = 0.066). There was also a main effect of cue validity (*F*(1,96) = 265.03, *p* ≤ 0.0001, η_p_^2^ = 0.73) but not cue valence (*F*(1,96) = 1.65, *p* = 0.20, η_p_^2^ = 0.017).

The analyses separating the anxiety groups were performed on the data from Experiment 1 after the mean trait anxiety scores were matched with those from Experiment 2. For the participants with higher trait anxiety, a two (cue validity) × two (cue valence) ANOVA revealed a significant main effect of validity (*F*(1,45) = 128.57, *p* < 0.0001, η_p_^2^ = 0.74) but not valence (*F* < 1). However, there was an interaction between validity and valence (*F*(1,45) = 8.42, *p* = 0.0058, η_p_^2^ = 0.16). Scheffé tests revealed that participants with higher trait anxiety responded faster after negatively valenced (angry) compared to neutral cues, but only when these cues were invalid (*p* < 0.036) and not when they were valid (*p* = 0.059). For the participants with lower trait anxiety, a two (cue validity) × two (cue valence) ANOVA revealed a significant main effect of validity (*F*(1,53) = 137.11, *p* < 0.0001, η_p_^2^ = 0.72), a marginally significant main effect of valence (*F*(1,53) = 3.98, *p* = 0.05, η_p_^2^ = 0.070), but no interaction between the two variables (*F* < 1).

### 3.3. Discussion

The findings of Experiment 2 were broadly consistent with the previous findings of Fox et al. (2002) and unlike those of Experiment 1, where stimulus emotionality was derived via learned semantic associations rather than directly from the visual features of the stimuli. Specifically, in invalid trials in Experiment 2, participants were slower to respond following an angry invalid cue than a neutral invalid cue, thus potentially reflecting a difficulty disengaging attention from the emotional stimuli. This pattern did not hold, nor reverse, for the valid trials. Notably, a reversal would have suggested a preferential orientation towards to the angry cues, rather than a difficulty disengaging from them. Consistent with previous findings with visually angry faces, this effect appeared to be driven by the participants with higher trait anxiety.

## 4. General Discussion

The findings reported here suggest that emotional knowledge about faces can impact visual processing mechanisms, specifically those involved in driving attentional orienting. Intriguingly, when compared to the effects of emotionality derived directly from the visual features of the face stimulus, evidence emerged of differences in the manner in which it does so. Specifically, in our studies, trait anxiety differentially impacted attention to negatively emotional stimuli as a function of whether the emotionality was acquired through semantic learning or derived directly from the visual stimulus. In Experiment 1, the participant group with higher trait anxiety showed *faster* responses following invalid faces cues when the faces had negative, compared to neutral, emotional knowledge, suggesting attentional avoidance of such stimuli. This same effect was not present for the participant group with lower trait anxiety. In contrast, in Experiment 2, the participant group with higher trait anxiety appeared to show increased attentional lingering on faces with angry expressions, relative to those with neutral expressions, as responses were *slower* after invalid face cues wearing angry facial expressions. This later finding is consistent with previous studies demonstrating difficulty disengaging from visually emotional stimuli by participants with higher anxiety [20].

An alternative account of the faster responses of the higher-trait-anxiety group following invalid faces cues with learned negative associations is that it might be a result of faster disengagement from, rather than avoidance of, these faces. This would suggest a more efficient processing of these stimuli by this group. However, this account would also predict faster responses following valid negative face cues, and the marginally significant *slowing* of responses in these trials is inconsistent with this account. Thus, the evidence seems more in line with the avoidance of face cues associated with negative emotional knowledge.

Placing the current findings in the context of the existing literature is challenging because of the variety of attentional biases that have been demonstrated for threat-related stimuli, as well as the variability in when, and under what conditions, these different biases are demonstrated [1]. The dot probe paradigm appears to be particularly sensitive to contextual factors that are not yet fully understood, questioning the ease with which the results can be interpreted and impacting its reliability and, thus, usefulness as an individual difference measure. Notably, the internal reliability of the bias to attend to faces with threatening expressions typically demonstrated in this task is greater when the probe (emotional stimulus) duration is short (i.e., 100 ms), compared to when it is longer (i.e., 300 ms or longer; [33]). Notably, even when short durations were used, the reliability of the effect was limited, suggesting that more research is needed to better understand other potential factors that impact attentional biases to emotional stimuli.

The variety of attentional biases and, more generally, the variability in the patterns of performance demonstrated for emotional stimuli are not as surprising when one considers that attention is not a unitary system and is better described as a complex network of mechanisms. Therefore, interpreting these findings requires consideration of not only the nature of the different attention biases and the stimulus and task factors that may impact them, but also the attentional mechanisms and their time courses.

There is evidence that the temporal aspects of the presentation of threatening stimuli are important in determining the nature of attentional bias. For example, Cooper and Langton [34] found an attentional bias towards the location of a relatively threatening stimulus after a 100 ms presentation, but this pattern reversed when the presentation duration was extended to 500 ms. Further, another study reported that a high-trait-anxiety group demonstrated an attention bias towards (prioritisation) threatening stimuli presented for 500 ms, but a bias away (avoidance) when they were presented for longer durations (i.e., 1250 ms) [28]. Interestingly, in that study, both the high- and low-trait-anxiety groups demonstrated prioritisation of threatening stimuli when presented for 100 ms. Zvielli and colleagues [35] speculated that relatively longer presentation durations may increase the probability that participants will demonstrate markers of both attentional prioritisation and avoidance, while relatively shorter presentation durations may only allow for the demonstration of attentional prioritisation.

While Experiment 2 found evidence of slower disengagement from visually threatening stimuli, consistent with prior studies using presentation durations between 100 and 500 ms (e.g., [20,23,31,36]), the findings of Experiment 1 using stimuli whose threatening nature was derived indirectly via semantic knowledge was more akin to what would be expected if the stimuli were presented for a longer duration. One possible explanation for the difference in attention patterns between Experiments 1 and 2 is that attentional holding effects (slower disengagement), such as that for the visually emotional stimuli in Experiment 2, may be driven by the rapidly accessed perceptual features of visually emotional stimuli. In contrast, avoidance biases typically observed after longer presentations, such as that observed in Experiment 1 for the semantically threatening faces, may be driven by the slower-to-access emotional meaning of stimuli.

Notably, existing findings within the literature suggest that some caution is necessary in generalising results from studies with high-trait-anxiety groups to clinically diagnosed groups. Specifically, while a high-trait-anxiety group showed the expected slower disengagement from faces wearing threat-related expressions (angry or fearful), a clinically diagnosed generalised anxiety disorder group showed the opposite pattern, that is, faster disengagement from threat-related faces [37]. Interestingly, this study also used a relatively long presentation duration, that is, 600 ms, which may have different consequences in the context of different manifestations of high anxiety (i.e., that is, when it meets or does not meet diagnostic criteria for an anxiety disorder). To better understand the roles of stimulus presentation duration and the clinical status of the anxiety of the sample tested, further studies are required. Given this difference in findings between a clinically diagnosed and a non-diagnosed high-trait-anxiety group, a future study examining the effect of learned negative associations on attention to visually neutral faces in a clinical sample would be worthwhile.

One account of the attentional biases in anxiety, known as the vigilance–avoidant hypothesis [38], may be particularly helpful for understanding the difference in the attentional biases observed for stimuli with threatening semantic associations compared to those that are visually threatening. This account proposes that anxiety is associated with an initial hypervigilance to threatening stimuli followed by a later avoidance response. The avoidance response is believed to be an effort to minimise discomfort arising from orienting to, and processing, the threatening stimuli. Thus, in the context of the vigilance–avoidant hypothesis, the findings of Experiment 1 could be interpreted as evidence that orienting to stimuli with negative semantic associations can produce a similar discomfort to orienting to visually emotional stimuli.

A recent study using stimuli with acquired emotional associations via classical conditioning found evidence consistent with the tendency for participants to avoid or suppress stimuli with acquired, non-perceptual, negative emotionality. Specifically, participants with higher anxiety appeared to rapidly engage mechanisms to inhibit the location where a conditioned threat stimulus appeared [39]. However, a previous behavioural study using the attentional disengagement paradigm found that visually neutral spatial cues (coloured squares) that had been conditioned with a loud burst of noise were associated with faster responding in valid trials and slower responding in invalid trials relative to non-conditioned cues [40]. Future research is needed to better tease apart these effects and to delineate the conditions under which stimuli associated with threat will capture, hold, or repel attention.

Notably, while much of the focus of this discussion has been on understanding the attentional bias observed in the higher anxiety group, it is important to note that the participants with lower anxiety were also impacted by the valence of the learned associations for the neutral faces in Experiment 1, but in a different manner than the group with high anxiety. Specifically, they showed a general response slowing, regardless of the validity of the face cue, for stimuli with negative, relative to neutral, semantic associations. Interestingly, a general response slowing for visually emotional stimuli has been documented in previous studies, but this is typically amongst participants with higher anxiety (e.g., [41]). Nonetheless, this finding demonstrates the broader impact of emotional knowledge about a stimulus on how it is processed.

Current theories of attentional biases in psychopathology postulate that individual differences in attention to emotional (threatening) stimuli stem from early evaluation of stimulus valence, as opposed to differences in the allocation of attentional resources [36,42]. The mechanisms through which this early evaluation system operates, however, remain unclear. The current study suggests that valence appraisals that are based on semantic knowledge of a stimulus’s emotional properties may be impacted differently by trait anxiety than are appraisals based on visually extracted emotional information. Given considerable interest in, and use of, attention bias modification interventions to treat anxiety [43], understanding the nature of the attention biases with respect to threat-related stimuli demonstrated by higher anxiety groups, as well as the factors that determine exactly what type of bias is likely to be exhibited, is important. However, the difference in the nature of the biases reported across previous studies, and the difference reported here for stimuli with visually versus semantically derived (threatening) emotionality, highlight the complexity of the nature of attentional biases demonstrated from emotional stimuli and their relationship with anxiety.

## Data Availability

The data presented in this study are available on request from the corresponding author.

## Appendix A

Trained Characteristics in Experiment 1.

**Negative:**
Angry, Violent, Unpredictable
Hateful, Selfish, Cruel
Merciless, Vicious, Deceitful
Irrational, Abusive, Demanding
**Neutral:**
Busy, Curious, Practical
Modest, Careful, Reserved
Calm, Disciplined, Confident
Thoughtful, Serious, Attentive.
